# Comparative efficacy and safety of dupilumab versus newly approved biologics and JAKi in pediatric atopic dermatitis: A systematic review and network meta-analysis

**DOI:** 10.1371/journal.pone.0319400

**Published:** 2025-02-24

**Authors:** Qiwei Liao, Hanwen Pan, Yixin Guo, Yuxiang Lan, Zhuo Huang, Peiyi Wu

**Affiliations:** Foshan Clinical Medical School of Guangzhou University of Chinese Medicine, Foshan, Guangdong, China; Mansheyet El Bakry General Hospital, EGYPT

## Abstract

**Background:**

The newly approved biologics and Janus kinase inhibitors (JAKi) for pediatric atopic dermatitis (AD) offer additional options for clinical treatment. However, the efficacy and safety differences compared to the first approved biologic, dupilumab, remain unclear. Therefore, a network meta-analysis was conducted to evaluate these differences and identify potentially superior agents.

**Methods:**

This systematic review was PROSPERO-registered (CRD42024583658). Randomized controlled trials involving pediatric patients (<18 years old) published in PubMed, Embase, Web of Science, and the Cochrane Library up to October 27, 2024 were searched and screened. RevMan software was utilized for quality assessment, and  meta-analysis was performed using R version 4.4.1. Efficacy measures included the Investigator’s Global Assessment (IGA), the Numeric Rating Scale for Itch (NRS), and the Eczema Area and Severity Index (EASI). The results of these measures were expressed as odds ratios (OR), while treatment rankings of different interventions were determined using the P-score.

**Result:**

This study included 11 trials involving 7 agents and 2,352 pediatric patients. The results indicated that dupilumab (300 mg) showed better outcomes than placebo in IGA-0/1 (OR =  4.68, 95% CI: 2.53–8.63), NRS-4 (OR =  6.75, 95% CI: 3.85–11.86), and all EASI outcomes. Tralokinumab may be the most effective option for alleviating pruritus (P-score for NRS-4, 0.8447). Upadacitinib (30 mg) performed best in IGA-0/1 (P-score, 0.9414), EASI-90 (P-score, 0.9926), and EASI-75 (P-score, 0.9707). Dupilumab (300 mg) had a higher risk of nasopharyngitis compared to placebo (OR = 2.15, 95%CI: 1.04–4.43). Compared to both placebo and dupilumab (300 mg), adverse event rates were higher with upadacitinib (15 mg and 30 mg), and upper respiratory tract infection risk was elevated with baricitinib (2 mg and 4 mg) and tralokinumab (300 mg).

**Conclusion:**

The efficacy of dupilumab for pediatric AD remains substantial, while other agents including upadacitinib, delgocitinib, and tralokinumab also present certain advantages. Future clinical trials may necessitate further evaluation of safety concerns.

## Introduction

Atopic dermatitis (AD) is a chronic, relapsing inflammatory skin disease characterized by itching and eczematous lesions, primarily associated with immune dysfunction [1]. It typically manifests during childhood, with a prevalence of 12.1% among children aged 6 months to 5 years and 14.8% among adolescents aged 12 to 17 years [2–5]. Standard treatments for pediatric AD currently include corticosteroids and calcineurin inhibitors [6–9]. However, patients with moderate-to-severe AD frequently encounter inadequate responses or intolerance to these therapeutic options [10]. Recent research indicates that AD is associated with immune abnormalities characterized by the abnormal production of cytokines such as IL-4, IL-5, IL-12, IL-13, and IFN-γ [11–14]. These cytokines trigger inflammatory responses by activating Janus kinase. Dupilumab, the first biologic agent approved by the Food and Drug Administration (FDA) in 2020 for the treatment of pediatric patients aged ≥ 6 with moderate-to-severe AD, targets the IL-4Rα subunit and inhibits inflammatory responses by blocking the signaling of IL-4 and IL-13 [15–18]. Tralokinumab and nemolizumab are also biologics that inhibit inflammatory responses by blocking interleukins. Additionally, upadacitinib, baricitinib, abrocitinib, and delgocitinib are Janus kinase inhibitors (JAKi) that effectively block the JAK/STAT pathway, leading to the inhibition of interleukin signaling [19–21]. Currently, biological therapy for pediatric AD is no longer restricted to dupilumab. Delgocitinib has been approved in Japan for the treatment of AD in children, while upadacitinib, abrocitinib, nemolizumab, and tralokinumab have received approval for adolescent AD [22]. However, due to the absence of direct head-to-head comparisons, the efficacy and safety differences between these new agents and dupilumab for pediatric AD remain unclear. Therefore, it is crucial to conduct a network meta-analysis (NMA) to evaluate these differences and identify superior treatment options.

## Materials and methods

### Protocol registration

This study adhered to the PRISMA guidelines, and the protocol was registered in PROSPERO (registration number CRD42024583658).

### Search strategy

Six researchers searched for literature from the inception of the databases to October 27, 2024 in PubMed, Embase, Web of Science, and the Cochrane Library. The following search terms were employed: “upadacitinib,” “dupilumab,” “baricitinib,” “abrocitinib,” “tralokinumab,” “delgocitinib,” “nemolizumab,” “lebrikizumab,” “Janus kinase inhibitors,” “JAK inhibitors,” “JAKi,” “biologics,” “monoclonal antibody,” “adolescents,” “children,” “pediatric,” and “atopic dermatitis.” The detailed search strategy is presented in Table S1 in S1 File.

### Inclusion criteria

This study is designed to include only randomized controlled trials (RCTs). The study population consists of pediatric patients under the age of 18 years diagnosed with atopic dermatitis, with no restrictions based on race or gender. Participants in the treatment group will receive either Janus kinase inhibitors (JAKi) or biologics, with or without additional topical therapies such as corticosteroids and antihistamines. The control group will receive either a placebo or a placebo combined with topical therapies.

### Exclusion criteria

1) Non-randomized controlled trials; 2) Trials on adult and adolescent patients without detailed reporting of baseline levels and outcomes for adolescents; 3) Outcome data for different doses of agents were combined for reporting.

### Literature screening and data extraction

Four researchers (QW. Liao, YX. Guo, PY. Wu, and Z. Huang) utilized Endnote X9 to remove duplicate literature. Following this, both the initial screening (based on title and abstract) and the secondary screening (full-text assessment) were conducted independently. Any literature with screening conflicts or uncertainties was meticulously documented, and decisions regarding inclusion or exclusion were reached through discussion. Articles that could not be conclusively resolved were addressed through discussions with HW. Pan and YX. Lan.

Subsequently, four researchers independently extracted the following information: first author, year of publication, trial registration number, treatments, patient demographics (including age, gender, and weight), disease duration, treatment duration, and efficacy and safety outcomes.

Efficacy outcomes included the percentage of individuals achieving an Investigator Global Assessment (IGA) score of 0 (clear) or 1 (almost clear) with a minimum improvement of ≥2 steps from baseline, or meeting the IGA response criteria defined in the studies (IGA 0/1); Peak Pruritus Numerical Rating Scale (PP-NRS), weekly Average Pruritus Numerical Rating Scale (AP-NRS) or Worst Pruritus Numerical Rating Scale (WP-NRS) with at least a 4-point improvement (NRS-4); and a ≥90% improvement in the Eczema Area and Severity Index score from baseline (EASI-90); as well as EASI-75; and EASI-50. Safety outcomes included adverse events (AEs), serious adverse events (SAEs), and occurrences of nasopharyngitis, upper respiratory tract infections (URTI), and conjunctivitis. Any inconsistencies encountered during the data extraction process were resolved through discussion.

### Quality assessment

The risk of bias in the included literature was assessed by four researchers using the Cochrane Collaboration Risk of Bias Tool. The quality assessment index comprised seven items: random sequence generation, allocation concealment, blinding of participants and personnel, blinding of outcome assessment, incomplete outcome data, selective reporting, and other bias. These items were categorized into three levels of risk: low risk of bias, unclear risk of bias and high risk of bias. The four researchers conducted independent evaluations and subsequently compared their findings. Any discrepancies that emerged were resolved through consultation with a fifth researcher.

### Statistical analysis

A network meta-analysis was conducted using the netmeta package in R version 4.4.1. The netmeta package, a frequentist-based tool for network meta-analysis, can serve as an alternative to Bayesian NMA package and has demonstrated reliability in previous network meta-analyses [23–25]. The heterogeneity of the data was assessed using Cochran’s Q. A fixed-effect model was employed when P >  0.1 and I² <  50%, while a random-effects model was applied if P <  0.1 or I² ≥  50%. All outcome measures were reported as odds ratios (OR) with corresponding 95% confidence intervals (95% CI) for interval estimation. A 95% CI that does not include 1 indicates a significant difference between the agent and dupilumab. Additionally, the P-score of the frequentist network meta-analysis estimate was utilized to rank interventions. The P-score, which is derived from point estimates and standard errors, reflects the degree of certainty that one treatment is superior to another. It serves a similar function to Bayesian SUCRA and produces comparable numerical values [26]. The higher the P-score, the better the intervention’s effectiveness. Furthermore, the “netsplit” command, based on the node-splitting approach, was utilized for consistency testing, allowing for assessment of discrepancies between direct and indirect evidence in the NMA [27].

## Result

### Study selection

**Fig 1:**
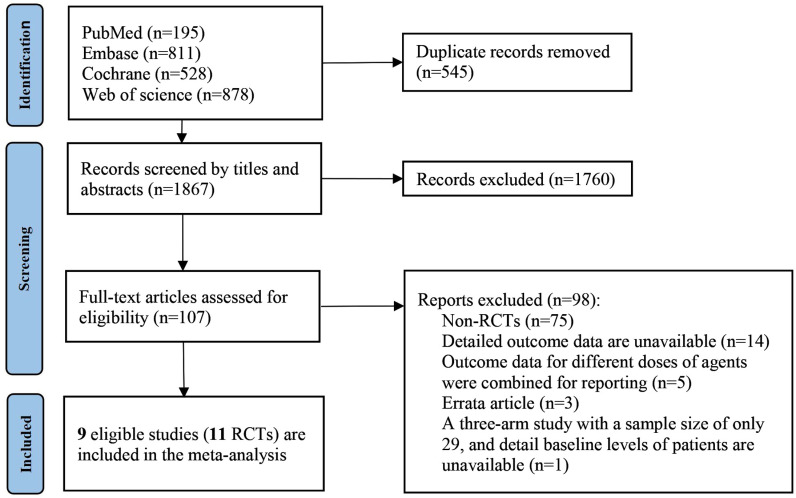
Flow chart of the search process.

A total of 2,412 articles were initially searched, with 545 duplicates removed using EndNote X9 software. Ultimately, 9 articles meeting inclusion criteria were selected, of which 8 reported on a single trial each. Notably, Paller’s study reported on 3 trials involving upadacitinib (Measure Up 1, Measure Up 2, and AD Up) [10]. Consequently, this study included a total of 2,352 patients from 11 RCTs (Fig 1).

### Study characteristics and quality assessment

Among the 11 RCTs, five trials investigated the combination of biologics (or JAKi) and topical therapies [10,28–31]. The topical therapies included corticosteroids, calcineurin inhibitors, antihistamines, and crisaborole. Six trials focused on monotherapy with biologics (or JAKi) [10,28,32–35]. Regarding treatment duration, NCT03796676 lasted 12 weeks, while JAPICCTI-173553 and JAPICCTI-184064 lasted 4 weeks [31,33,34]. The remaining trials had a duration of 16 weeks [10,28–30,32,35]. The mean baseline Eczema Area and Severity Index (EASI) score for patients was 26.3. Additionally, excluding JAPICCTI-173553 and JAPICCTI-184064, which recruited patients with mild atopic dermatitis (IGA ≤  2), the other trials enrolled patients with moderate-to-severe AD (IGA ≥  3) [33,34,36]. Detailed study characteristics are provided in Table 1.

**Table 1:** Study characteristics of the included studies.

Study	Registration number	Week of eveluation of response,w	Treatment*	Topical therapy	Sample size		Age, y(mean)		Male/Female		Disease duration, y(mean)		Basal EASI score (Mean)		IGA ≥ 4, n	
					Treat	Control	Treat	Control	Treat	Control	Treat	Control	Treat	Control	Treat	Control
Paller 2020	NCT03345914	16	Dupilumab 300mg q4w	corticosteroids	122	123	8.5	8.3	57/68	61/62	7.4	7.2	37.4	39	NA	NA
Simpson 2020	NCT03054428	16	Dupilumab 300mg q4w	NA	84	85	14.4	14.5	52/32	53/32	11.9	12.3	35.8	35.5	46	46
Torrelo 2023	NCT03952559	16	Baricitinib 1mg qd	corticosteroids	121	122	12.4	11.8	59/62	58/64	9.8	9.2	26.6	27	45	48
	NCT03952559	16	Baricitinib 2mg qd	corticosteroids	120	122	11.8	11.8	57/63	58/64	9.4	9.2	26.8	27	46	48
	NCT03952559	16	Baricitinib 4mg qd	corticosteroids	120	122	11.9	11.8	67/53	58/64	9	9.2	25.3	27	45	48
Igarashi 2024	JRCT2080225289	16	Nemolizumab 30mg q4w	corticosteroids or calcineurin inhibitors or Antihistamines	45	44	9.2	8.9	29/16	24/20	8.9	8	19.8	16.2	9	10
Eichenfield 2021	NCT03796676	12	Abrocitinib 100mg qd	corticosteroids or calcineurin inhibitors or crisaborole	89	94	16	14	45/50	44/52	9.8	10.5	31	29.2	38	39
	NCT03796676	12	Abrocitinib 200mg qd	corticosteroids or calcineurin inhibitors or crisaborole	93	94	15	14	56/38	44/52	9.7	10.5	29.5	29.2	33	39
Paller 2023	NCT03568318	16	Upadacitinib 15mg qd	corticosteroids	60	63	15.4	15.1	30/30	27/36	11.4	12.3	29.6	30.3	31	35
	NCT03568318	16	Upadacitinib 30mg qd	corticosteroids	60	63	15.3	15.1	28/32	27/36	12.2	12.3	28.7	30.3	31	35
	NCT03569293	16	Upadacitinib 15mg qd	NA	64	61	15.5	15.1	30/34	28/33	12	11.4	30.7	29.7	29	26
	NCT03569293	16	Upadacitinib 30mg qd	NA	64	61	15.7	15.1	28/26	28/33	12.4	11.4	27.8	29.7	27	26
	NCT03607422	16	Upadacitinib 15mg qd	NA	58	60	15.2	15.5	20/42	25/35	11.2	12.2	28	30.1	31	34
	NCT03607422	16	Upadacitinib 30mg qd	NA	62	60	15.8	15.5	36/22	25/35	12.1	12.2	31.2	30.1	33	34
Paller 2023	NCT03526861	16	Tralokinumab 150mg q2w	NA	98	94	15	14	51/47	51/43	13	13	28.9	27.2	44	43
	NCT03526861	16	Tralokinumab 300mg q2w	NA	97	94	15	14	47/50	51/43	13	13	28	27.2	48	43
Nakagawa 2019	JAPICCTI-173553	4	Delgocitinib 0.25% bid	NA	34	35	8.4	8.6	12/22	18/17	6.1	6.4	10.45	11.25	1	2
	JAPICCTI-173553	4	Delgocitinib 0.5% bid	NA	34	35	8.5	8.6	18/16	18/17	6.6	6.4	11.11	11.25	1	2
Nakagawa 2021	JAPICCTI-184064	4	Delgocitinib 0.25% bid	NA	69	68	8.2	8.3	39/30	31/37	5.8	6.2	10.7	10.6	16	14

* q4w =  every four weeks, q2w =  every two weeks, qd =  once daily, bid =  twice daily. NA =  Not Available.

**Fig 2:**
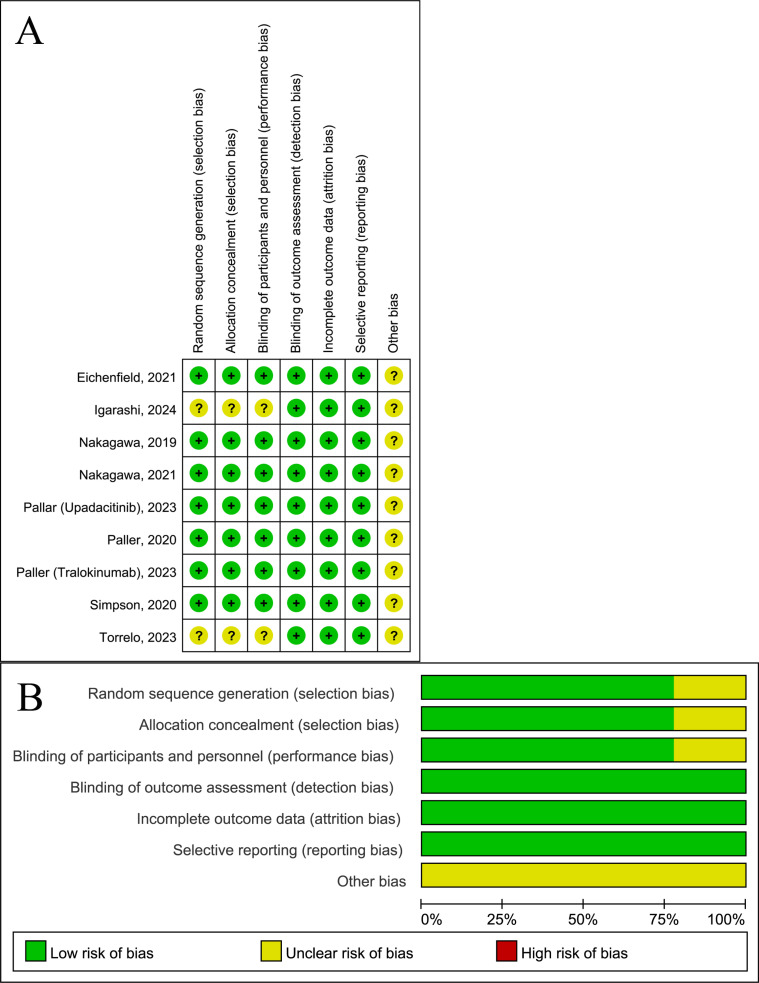
Quality assessment of the included studies. (A) Risk of bias graph (B) Risk of bias summary.

Only two studies, NCT03952559 and JRCT2080225289, did not provide detailed information regarding random sequence generation, allocation concealment, and blinding of participants and personnel [29,30]. The remaining literature exhibited only uncertainties related to “other biases,” and the overall quality of the included studies was deemed acceptable (Fig 2).

### Assessment of heterogeneity and consistency

The heterogeneity analysis for each outcome is presented in Table S2 in S1 File. Moderate heterogeneity was observed for the EASI-90 outcome (I^2^ =  24.9%), while the heterogeneity for the remaining outcomes was low. Consequently, a fixed-effects model was employed for both efficacy and safety outcomes. Given the absence of direct comparisons between different agents, we performed a consistency test for varying doses of the same agent. The results indicated no significant differences between the outcomes of direct and indirect comparisons.

### Efficacy comparison

**Fig 3:**
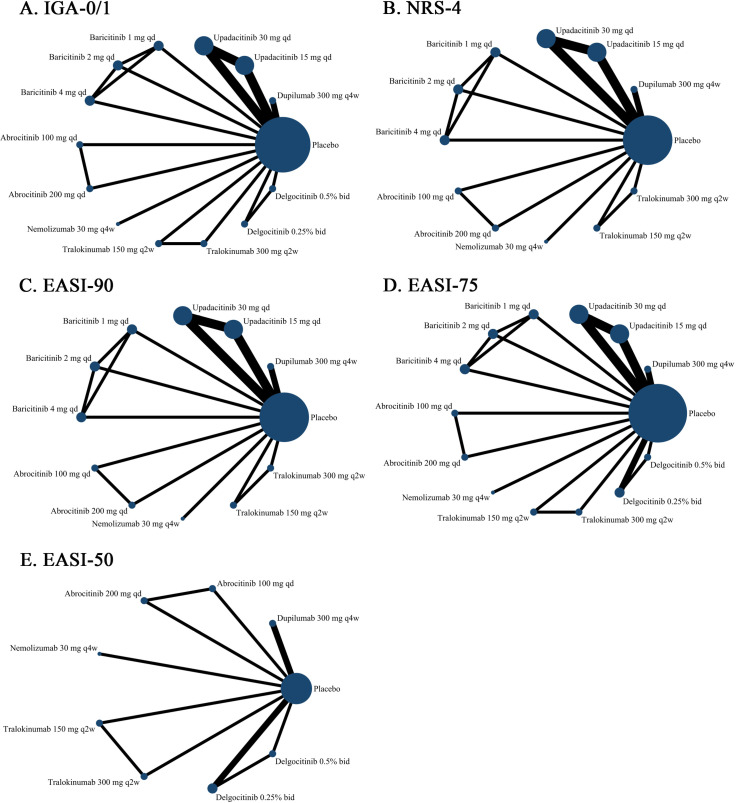
Network diagram of efficacy outcomes. The width of the lines represents the number of RCTs, and the size of the nodes represents the sample size.

The network relationships among the various interventions are illustrated in Fig 3. In comparison to the placebo, dupilumab (300 mg) demonstrated superior efficacy across all efficacy outcome measures (Fig S1 in S1 File). According to the P-score ranking results, dupilumab (300 mg) is ranked 6th in IGA-0/1 (P-score, 0.5904), 5th in NRS-4 (P-score, 0.7101), 2nd in EASI-90 (P score, 0.8322), 5th in EASI-75 (P-score, 0.6680), and 1st in EASI-50 (P-score, 0.9776) (Tables S8–S12 in S1 File).

**Fig 4:**
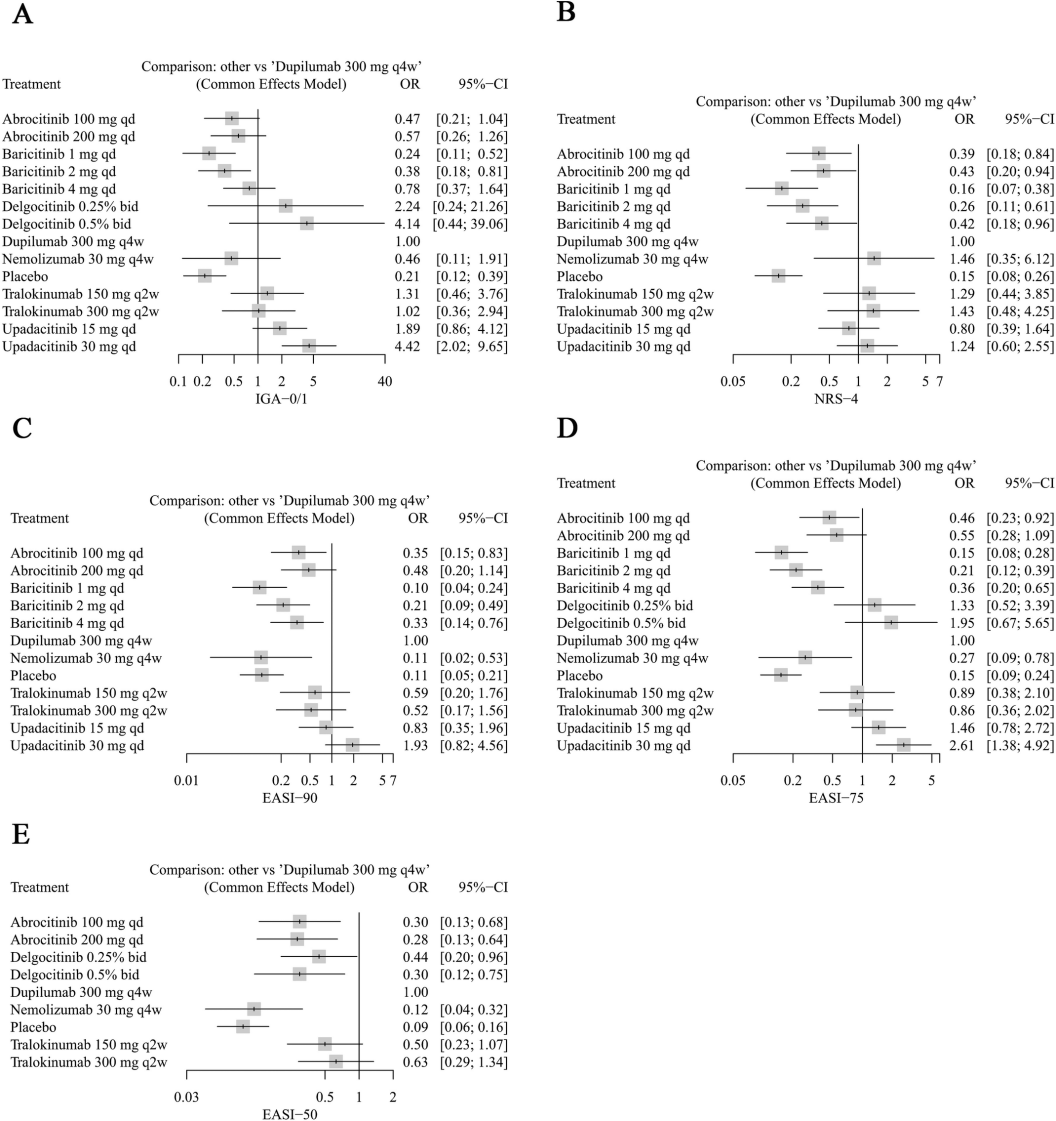
Forest plots of efficacy outcomes (comparison with dupilumab 300 mg q4w).

In comparison to dupilumab (300 mg), only upadacitinib (30 mg) exhibited superior efficacy among JAKi, with an OR of 4.42 (95% CI: 2.02–9.65) for IGA-0/1 and an OR of 2.61 (95% CI: 1.38–4.92) for EASI-75 (Fig 4A and 4D). Furthermore, upadacitinib (30 mg) ranked 1st in IGA-0/1 (P-score, 0.9414), EASI-90 (P-score, 0.9926), and EASI-75 (P-score, 0.9707), demonstrating prominent therapeutic advantages (Tables S8, S10 and S11 in S1 File). Delgocitinib (0.5% and 0.25%) exhibited inferior performance relative to dupilumab (300 mg) in achieving EASI-50 (OR =  0.44, 95% CI: 0.20-0.96 and OR =  0.28, 95% CI: 0.13–0.64) (Fig 4E). Nonetheless, it showed a trend towards superior performance compared to dupilumab in the P-score rankings for IGA-0/1 (P-score, 0.8827 and 0.7380) and EASI-75 (P-score, 0.8850 and 0.7613) (Tables S8 and S11 in S1 File). The efficacy of baricitinib and abrocitinib is not particularly remarkable, as the P-scores for all outcome measures rank below those of dupilumab (300 mg) (Tables S8–S12 in S1 File). However, both abrocitinib (100 mg and 200 mg) and baricitinib (4 mg) still demonstrated a significant improvement over placebo (Fig S1 in S1 File).

In terms of biologics, the efficacy of tralokinumab (150 mg and 300 mg) is comparable to that of dupilumab (300 mg) (Fig 4), with a tendency for superior performance in NRS-4 (P-scores 0.8447 and 0.7988) (Table S9 in S1 File). Nemolizumab (30 mg) exhibited inferior performance relative to dupilumab (300 mg) in EASI-90 (OR =  0.11, 95% CI: 0.02–0.53), EASI-75 (OR =  0.27, 95% CI: 0.09–0.78), and EASI-50 (OR =  0.12, 95% CI: 0.04–0.32) (Fig 4C, 4D and 4E). However, it showed a tendency towards better performance in the ranking of P-score for NRS-4 (P-score, 0.8194) (Table S9 in S1 File).

### Safety comparison

**Fig 5:**
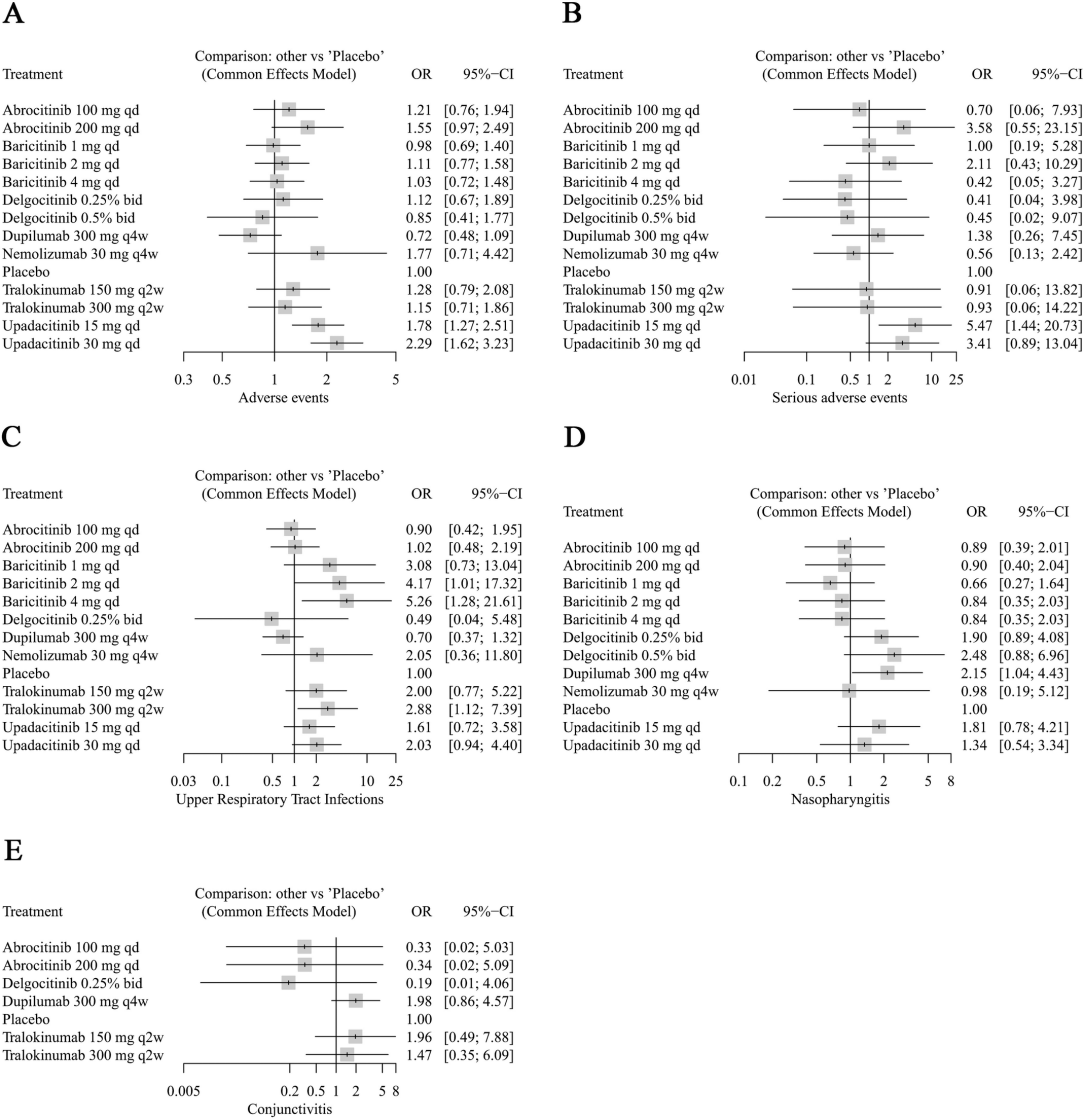
Forest plots of safety outcomes (comparison with placebo).

Compared to placebo, the incidence of adverse events (AEs) was higher with upadacitinib (30 mg) (OR =  2.29, 95% CI: 1.62–3.23). Additionally, the incidence of AEs (OR =  1.78, 95% CI: 1.27–2.51) and serious adverse events (SAEs) (OR =  5.47, 95% CI: 1.44–20.73) was elevated with upadacitinib (15 mg) (Fig 5A and 5B). Baricitinib (1 mg) (OR =  5.47, 95% CI: 1.44–20.73), baricitinib (2 mg) (OR =  5.47, 95% CI: 1.44–20.73), and tralokinumab (300 mg) (OR =  5.47, 95% CI: 1.44–20.73) had a higher risk of upper respiratory tract infections (URTI) (Fig 5C), while dupilumab (300 mg) was linked to an increased risk of nasopharyngitis (OR =  2.15, 95% CI: 1.04–4.43) (Fig 5D).

Furthermore, in comparison to dupilumab (Fig S2 in S1 File), the incidence of AEs was also higher with upadacitinib (15 mg) (OR =  2.46, 95% CI: 1.44–4.20) and upadacitinib (30 mg) (OR =  3.16, 95% CI: 1.85–5.40) (Fig S2A in S1 File), while the risk of URTI was higher with baricitinib (1 mg) (OR =  5.97, 95% CI: 1.26–28.40), baricitinib (2 mg) (OR =  7.53, 95% CI: 1.60–35.47), and tralokinumab (300 mg) (OR =  4.12, 95% CI: 1.32–12.85) (Fig S2C in S1 File).

### Sensitivity analysis

To investigate the potential impact of topical therapies (TT), such as corticosteroids, on the outcomes, a sensitivity analysis was conducted. The trials were categorized into combined therapy group and monotherapy group, based on whether topical therapies were used in conjunction with other treatments. The results of the sensitivity analysis were generally consistent with the original analysis (Figs S3–S6 in S1 File).

## Discussion

Dupilumab is the first biologic agent approved in 2020 for patients aged ≥6 years with moderate-to-severe AD [22,37]. Before its approval, pediatric patients with severe AD had limited options for systemic treatment, primarily relying on immunosuppressive drugs such as cyclosporine and azathioprine. However, the long-term administration of these medications has been limited by high discontinuation rates [38–41]. In contrast, the long-term discontinuation rates for dupilumab are low, highlighting the advantages of biological therapy [42]. Currently, several newly approved agents offer more options for the treatment of pediatric AD. To investigate potentially superior treatment options, we employed NMA to evaluate the comparative efficacy and safety of 6 newly approved agents versus dupilumab in this study.

Among the JAKi, only upadacitinib demonstrated significantly superior efficacy compared to dupilumab. This finding diverges from previous studies involving adult patients, where both abrocitinib and upadacitinib have exhibited superior efficacy relative to dupilumab [40,43,44]. But it is important to note that abrocitinib was included in only 1 study, which may influence the results. As of 2024, both upadacitinib and abrocitinib have received approval for use in patients aged ≥12 years in the United States and Canada [15]. Future head-to-head trials in pediatric AD may yield further therapeutic insights.

In March 2021, delgocitinib was approved for use in pediatric patients in Japan [16]. In this study, delgocitinib (0.25% and 0.5%) demonstrated comparable efficacy to dupilumab (300 mg) during short-term use. Furthermore, delgocitinib exhibited good efficacy and tolerability in both long-term and infantile treatment, establishing it as recommended therapeutic option [34,45]. Baricitinib has not been approved for the treatment of AD in North America; however, it is approved for patients aged ≥2 years with moderate-to-severe AD in Europe [22]. Although baricitinib does not exhibit the same efficacy as dupilumab, the higher doses of baricitinib (2 mg and 4mg) have demonstrated superior therapeutic effects compared to placebo. Therefore, it remains a viable treatment option.

In terms of biologics, tralokinumab (300 mg) and nemolizumab (30 mg) rank 1st and 3rd, respectively, in P-score for NRS-4, both demonstrating significant improvements in pruritus relief. Furthermore, tralokinumab (150 mg and 300 mg) exhibits comparable efficacy to dupilumab (300 mg) across other efficacy outcomes, which may be attributed to their shared mechanism of blocking IL-13 [32]. Conversely, nemolizumab targets IL-31, a cytokine directly associated with the development of pruritus in AD [46–48]. However, nemolizumab (30 mg) did not demonstrate significant improvements in EASI-related outcomes (Fig S1 in S1 File). This lack of efficacy may clarify why nemolizumab was prominently highlighted for the treatment of pruritus upon its approval for adolescents in Japan in 2022 [49].

In terms of safety outcomes, upadacitinib exhibited a higher incidence of AEs, with acne being the most prevalent (incidence rate, 51/361); however, only one patient discontinued treatment due to moderate acne. The occurrence of acne is typically thought to be related to follicular keratosis, and JAKi can interfere with the JAK/STAT pathway, leading to aberrant follicular keratinization [50,51]. Among the SAEs associated with upadacitinib (incidence rate, 4/361), only one case of grade 3 impetigo resulted in treatment discontinuation, while the other SAEs were deemed unrelated to the treatment. In assessing safety, we also evaluated nasopharyngitis, URTI, and conjunctivitis, considering their high incidence during biological therapies. While the incidence rates of nasopharyngitis with dupilumab (300 mg) and URTI with tralokinumab (300 mg) and baricitinib (2 mg and 4 mg) are relatively high, these reactions are generally not severe, do not necessitate discontinuation, and can be managed with symptomatic treatment. Based on data from the 11 pediatric trials included in this study, short-term biological therapies (4–16 weeks) demonstrated safety in pediatric patients consistent with observations in adult patients, and showed a favorable benefit-risk ratio.

As AD is a chronic condition, it is also essential to assess the efficacy and safety of long-term treatment. During the open-label extension phase of the trials which lasted 52–76 weeks, the efficacy observed during short-term treatment in the treatment group was typically maintained. Regarding safety, the most commonly reported AEs for upadacitinib (15 mg and 30 mg) continue to be acne. AEs associated with other agents are predominantly mild to moderate in severity and rarely result in treatment discontinuation. Notably, there were no reports of pulmonary embolism, deep vein thrombosis, arterial thrombosis, major adverse cardiovascular events, or malignancies [32,34,52–56].

To our knowledge, this study is the first network meta-analysis investigating the use of biological therapies in the treatment of pediatric AD. Furthermore, all included studies were high-quality RCTs with a low risk of bias. In contrast to the meta-analysis conducted by Santos et al., our study incorporated JAKi and conducted a comprehensive assessment of the efficacy and safety of 6 newly approved agents in comparison to dupilumab [57]. Consequently, this study may serve as a valuable reference for selecting more effective treatments and can assist pediatric AD patients who do not achieve clinical response with topical therapies or even dupilumab.

However, our study has several limitations that warrant consideration. Firstly, due to the long-term treatments being non-randomized, open-label trials without placebo control, we could only provide a brief descriptive analysis of the long-term treatment outcomes. Future RCTs focusing on long-term treatment may provide more robust quantitative analytical data. Secondly, due to the rarity of head-to-head comparison trials, our NMA is limited to constructing indirect comparisons. Differences in trail design and patient baseline characteristics may lead to discrepancies between study results and clinical reality. Finally, due to the inclusion of only 1 study on abrocitinib, we were unable to elucidate why its efficacy compared to dupilumab differed in adults. In the sensitivity analysis (Fig S3 in S1 File), abrocitinib (from NCT03796676) was evaluated against dupilumab (from NCT03054428). However, results remained unchanged even when all patients were treated with monotherapy, and there was further reduction in differences in patient characteristics (age, number, and baseline). Upon careful consideration, we tentatively attributed this discrepancy to the shorter treatment duration in the abrocitinib trial (12 weeks vs. 16 weeks). We anticipate that future head-to-head trials will provide further evidence to confirm or refute this finding. Notably, the other conclusions of our study are largely consistent with previous research involving adult patients [43,58]. Therefore, our results are still reliable and could offer valuable insights to this field.

## Conclusion

In summary, current evidence indicates that upadacitinib (30 mg) is the most effective biological therapy for treating adolescent AD. Delgocitinib (0.25% and 0.5%) and tralokinumab (150 mg and 300 mg) demonstrate efficacy comparable to that of dupilumab (300 mg). Nemolizumab (30 mg) offers certain advantages in alleviating pruritus. Moreover, the safety profiles of the 7 agents included in this study for pediatric AD are comparable to those observed in adult AD. The favorable efficacy-risk ratio of biologics and JAKi indicates that they can provide valuable support in the treatment of pediatric AD.

## Supporting information

S1 File Basic supporting information.
**Table S1.** Search Strategy. **Table S2.** Heterogeneity assessment. **Table S3–S7.** Network meta-analysis in comparing different interventions. **Table S8–S12.** P-score rankings results. **Fig S1.** Forest plots of efficacy outcomes (comparison with placebo). **Fig S2.** Forest plots of safety outcomes (comparison with dupilumab 300 mg q4w). **Fig S3–S6** Forest plots of sensitivity analysis.
(DOCX)

S2 File Full-text articles assessed for eligibility.
(XLSX)

S3 File Records excluded by title or abstract.
(XLSX)

S4 File Data extraction table.
(XLSX)

S5 File Quality assessment of the included studies.
(XLSX)

S6 File PRISMA Checklist.
(DOCX)

## Abbreviations

AEs: adverse events
SAEs: serious adverse events
URTI: upper respiratory tract infections
AD: atopic dermatitis
JAK: Janus Kinase
SUCRA: surface under the cumulative ranking
OR: odds ratios
RCTs: randomized controlled trials
qd: once a day
bid: twice a day
qw: every week
q2w: every 2 weeks
q4w: every 4 weeks
