# Supplementary material for: Comparative efficacy and safety of dupilumab versus newly approved biologics and JAKi in pediatric atopic dermatitis: A systematic review and network meta-analysis
Source: PLoS One. 2025 Feb 24;20(2):e0319400. doi: 10.1371/journal.pone.0319400 (PMC11849894; doi:10.1371/journal.pone.0319400)

***Supporting Information***

**Table S1. Search strategy.**

| **1.1. Search strategy in PubMed and Cochrane library** |
| --- |
| **#1** Upadacitinib OR Dupilumab OR Baricitinib OR Abrocitinib OR Tralokinumab OR Delgocitinib OR Lebrikizumab OR Nemolizumab OR Janus Kinase Inhibitors OR JAK Inhibitors OR JAKi OR Biologics OR Biological Products OR monoclonal antibody OR Monoclonal Antibodies  **#2** Adolescent* OR Children OR pediatric OR paediatric  **#3** Atopic dermatitis OR Atopic Neurodermatitis OR Atopic Eczema OR Infantile Eczema  **#4** randomized controlled trial OR randomized controlled trials OR RCT OR controlled clinical trial OR controlled clinical trials OR clinical trial OR clinical trials OR clinical study OR clinical studies OR randomly allocated OR random allocation OR randomized OR randomised OR randomization OR double-blind OR ((double OR treble OR triple) AND (mask* OR blind*))  **#5** #1 AND #2 AND #3 AND #4 |
| **1.2. Search strategy in Web of science** |
| **#1** Upadacitinib OR Dupilumab OR Baricitinib OR Abrocitinib OR Tralokinumab OR Delgocitinib OR Lebrikizumab OR Nemolizumab OR Janus Kinase Inhibitors OR JAK Inhibitors OR JAKi OR Biologics OR Biological Products OR monoclonal antibody OR Monoclonal Antibodies  **#2** Adolescent* OR Children OR pediatric OR paediatric  **#3** Atopic dermatitis OR Atopic Neurodermatitis OR Atopic Eczema OR Infantile Eczema  **#4** randomized controlled trial OR randomized controlled trials OR RCT OR controlled clinical trial OR controlled clinical trials OR clinical trial OR clinical trials OR clinical study OR clinical studies OR randomly allocated OR random allocation OR randomized OR randomised OR randomization OR double-blind OR ((double OR treble OR triple) AND (mask* OR blind*))  **#5** #1 AND #2 AND #3 AND #4  **Refined By: Not Document Types: Review article** |
| **1.3. Search strategy in Embase** |
| **#1** 'Upadacitinib' OR 'Dupilumab' OR 'Baricitinib' OR 'Abrocitinib' OR 'Tralokinumab' OR 'Delgocitinib' OR 'Lebrikizumab' OR 'Nemolizumab' OR 'Janus Kinase Inhibitors' OR 'JAK Inhibitors' OR 'JAKi' OR 'Biologics' OR 'Biological Products' OR 'monoclonal antibody' OR 'Monoclonal Antibodies':ab,kw,ti  **#2** 'Adolescent' OR 'adolescents' OR 'Children' OR 'pediatric' OR 'paediatric':ab,kw,ti  **#3** 'Atopic dermatitis' OR 'Atopic Neurodermatitis' OR 'Atopic Eczema' OR 'Infantile Eczema':ab,kw,ti  **#4** 'randomized controlled trial' OR 'randomized controlled trials' OR 'RCT' OR 'controlled clinical trial' OR 'controlled clinical trials' OR 'clinical trial' OR 'clinical trials' OR 'clinical study' OR 'clinical studies' OR 'randomly allocated' OR 'random allocation' OR 'randomized' OR 'randomised' OR 'randomization' OR 'double-blind' OR (('double' OR 'treble' OR 'triple') AND ('mask*' OR 'blind*')):ab,kw,ti  **#5** #1 AND # 2 AND #3 AND #4 AND ([article]/lim OR [article in press]/lim) |

**Table S2. Heterogeneity assessment.**

| **Outcome** | **Tau** | **I^2^** | **Q value - total** | **p value (Q test) - total** | **Q value - within designs** | **p value**  **(Q test) – within designs** | **Q value – between designs** | **p value (Q test) - between designs** |
| --- | --- | --- | --- | --- | --- | --- | --- | --- |
| **Efficacy outcomes** | | | | | | | | |
| IGA-0/1 | 0 | 0% | 6.43 | 0.9544 | 6.41 | 0.4923 | 0.02 | 1.0000 |
| NRS-4 | 0.1086 | 5.6% | 13.77 | 0.3904 | 13.73 | 0.0562 | 0.04 | 1.0000 |
| EASI-90 | 0.2523 | 24.9% | 17.31 | 0.1857 | 17.16 | 0.0164 | 0.15 | 0.9999 |
| EASI-75 | 0 | 0% | 13.72 | 0.5466 | 13.42 | 0.0982 | 0.30 | 0.9999 |
| EASI-50 | 0 | 0% | 0.86 | 0.9732 | 0.86 | 0.6518 | 0.00 | 1.0000 |
| **Safety outcomes** | | | | | | | | |
| AEs^1^ | 0 | 0% | 4.32 | 0.9965 | 3.63 | 0.8891 | 0.69 | 0.9984 |
| SAEs^2^ | 0 | 0% | 1.08 | 1.0000 | 1.07 | 0.9977 | 0.01 | 1.0000 |
| Nasopharyngitis | 0 | 0% | 6.42 | 0.9547 | 6.37 | 0.6056 | 0.05 | 1.0000 |
| URTI^3^ | 0 | 0% | 9.50 | 0.7340 | 9.47 | 0.2208 | 0.04 | 1.0000 |
| Conjunctivitis | 0 | 0% | 0.2 | 0.9773 | 0.2 | 0.6534 | 0.00 | 1.0000 |

^1^ AEs = Adverse events

^2^ SAEs = Serious adverse events

^3^ URTI = Upper respiratory tract infection

**Table S3. Network meta-analysis in comparing different interventions for IGA-0/1.**

| Abrocitinib 100 mg | 1.21(0.67-2.17) | . | . | . | . | . | . | . | 0.46(0.24-0.86) | . | . | . | . |
| --- | --- | --- | --- | --- | --- | --- | --- | --- | --- | --- | --- | --- | --- |
| 1.21(0.74-1.97) | Abrocitinib 200 mg | . | . | . | . | . | . | . | 0.38(0.20-0.70) | . | . | . | . |
| 0.52(0.26-1.02) | 0.43(0.22-0.84) | Baricitinib 1 mg | 1.57(0.85-2.90) | 3.21(1.79-5.78) | . | . | . | . | 0.88(0.45-1.72) | . | . | . | . |
| 0.81(0.41-1.58) | 0.67(0.34-1.30) | 1.57(1.02-2.41) | Baricitinib 2 mg | 2.05(1.19-3.54) | . | . | . | . | 0.56(0.30-1.06) | . | . | . | . |
| 1.66(0.85-3.22) | 1.37(0.71-2.66) | 3.21(2.11-4.90) | 2.05(1.37-3.08) | Baricitinib 4 mg | . | . | . | . | 0.27(0.15-0.50) | . | . | . | . |
| 4.77(0.52-44.09) | 3.95(0.43-36.45) | 9.24(1.01-84.38) | 5.90(0.65-53.70) | 2.88(0.32-26.14) | Delgocitinib 0.25% | 1.85(0.52-6.63) | . | . | 0.10(0.00-1.85) | . | . | . | . |
| 8.82(0.96-80.99) | 7.29(0.79-66.95) | 17.09(1.88; 154.97) | 10.90(1.20-98.62) | 5.32(0.59-48.01) | 1.85(0.55-6.27) | Delgocitinib 0.5% | . | . | 0.05(0.00-0.94) | . | . | . | . |
| 2.13(0.96-4.72) | 1.76(0.80-3.90) | 4.13(1.93-8.82) | 2.63(1.24-5.59) | 1.28(0.61-2.71) | 0.45(0.05-4.24) | 0.24(0.026-2.28) | Dupilumab 300 mg | . | 0.21(0.12-0.39) | . | . | . | . |
| 0.98(0.25-3.90) | 0.81(0.21-3.23) | 1.91(0.49-7.41) | 1.22(0.31-4.71) | 0.59(0.15-2.29) | 0.21(0.02-2.55) | 0.11(0.01-1.37) | 0.46(0.11-1.91) | Nemolizumab 30 mg | 0.46(0.13-1.66) | . | . | . | . |
| 0.46(0.27-0.76) | 0.38(0.23-0.62) | 0.88(0.56-1.38) | 0.56(0.36-0.87) | 0.27(0.18-0.42) | 0.10(0.01-0.83) | 0.05(0.01-0.45) | 0.21(0.12-0.39) | 0.46(0.13-1.66) | Placebo | 6.14(2.02-18.65) | 4.78(1.54-14.80) | 8.58(4.58-16.09) | 21.25 (11.29-39.97) |
| 2.79(1.03-7.55) | 2.31(0.86-6.23) | 5.41(2.06-14.22) | 3.45(1.32-9.02) | 1.68(0.65-4.38) | 0.59(0.06-6.01) | 0.32(0.03-3.23) | 1.31(0.46-3.76) | 2.84(0.61-13.23) | 6.14(2.61-14.42) | Tralokinumab 150 mg | 0.78(0.38-1.59) | . | . |
| 2.18(0.80-5.90) | 1.80(0.67-4.87) | 4.22(1.60-11.12) | 2.69(1.03-7.05) | 1.31(0.50-3.42) | 0.46(0.04-4.69) | 0.25(0.02-2.52) | 1.02(0.36-2.94) | 2.21(0.47-10.33) | 4.78(2.03-11.28) | 0.78(0.41-1.49) | Tralokinumab 300 mg | . | . |
| 4.02(1.99-8.11) | 3.33(1.65-6.69) | 7.79(4.03-15.08) | 4.97(2.59-9.54) | 2.42(1.27-4.62) | 0.84(0.09-7.75) | 0.46(0.05-4.17) | 1.89(0.86-4.12) | 4.08(1.04-16.06) | 8.83(5.44-14.32) | 1.44(0.54-3.84) | 1.85(0.69-4.95) | Upadacitinib 15 mg | 2.31(1.52-3.51) |
| 9.40(4.66-18.97) | 7.78(3.86-15.66) | 18.22(9.41-35.28) | 11.62(6.05-22.32) | 5.67(2.97-10.81) | 1.97(0.21-18.13) | 1.07(0.12-9.75) | 4.42(2.02-9.65) | 9.55(2.43-37.56) | 20.65 (12.72-33.53) | 3.37(1.26-8.99) | 4.32(1.61-11.58) | 2.34(1.60-3.42) | Upadacitinib 30 mg |

**Table S4. Network meta-analysis in comparing different interventions for NRS-4.**

| Abrocitinib 100 mg | 0.8943(0.47-1.70) | . | . | . | . | . | 2.62(1.37-5.02) | . | . | . | . |
| --- | --- | --- | --- | --- | --- | --- | --- | --- | --- | --- | --- |
| 0.89(0.53-1.52) | Abrocitinib 200 mg | . | . | . | . | . | 2.93(1.52-5.64) | . | . | . | . |
| 2.43(1.05-5.60) | 2.71(1.17-6.27) | Baricitinib 1 mg | 0.6082(0.26-1.44) | 0.38(0.17-0.88) | . | . | 1.08(0.41-2.84) | . | . | . | . |
| 1.48(0.65-3.36) | 1.65(0.72-3.76) | 0.61(0.33-1.12) | Baricitinib 2 mg | 0.63(0.29-1.37) | . | . | 1.78(0.71-4.43) | . | . | . | . |
| 0.93(0.41-2.11) | 1.04(0.46-2.36) | 0.38(0.21-0.70) | 0.63(0.35-1.13) | Baricitinib 4 mg | . | . | 2.81(1.16-6.80) | . | . | . | . |
| 0.39(0.18-0.84) | 0.43(0.20-0.94) | 0.16(0.07-0.38) | 0.26(0.11-0.61) | 0.42(0.18-0.96) | Dupilumab 300 mg | . | 6.75(3.85-11.86) | . | . | . | . |
| 0.27(0.06-1.10) | 0.30(0.07-1.24) | 0.11(0.03-0.48) | 0.18(0.04-0.78) | 0.29(0.07-1.23) | 0.69(0.16-2.88) | Nemolizumab 30 mg | 9.84(2.6-36.82) | . | . | . | . |
| 2.62(1.54-4.45) | 2.93(1.72-4.99) | 1.08(0.57-2.07) | 1.78(0.95-3.34) | 2.81(1.51-5.23) | 6.75(3.85-11.86) | 9.84(2.63-36.82) | Placebo | 0.11(0.03-0.40) | 0.10(0.03-0.36) | 0.18(0.10-0.32) | 0.12(0.07-0.22) |
| 0.30(0.10-0.88) | 0.34(0.11-0.98) | 0.12(0.04-0.39) | 0.20(0.07-0.63) | 0.32(0.10-0.99) | 0.77(0.26-2.30) | 1.13(0.22-5.67) | 0.11(0.05-0.29) | Tralokinumab 150 mg | 0.90(0.47-1.76) | . | . |
| 0.27(0.09-0.79) | 0.30(0.10-0.89) | 0.11(0.04-0.35) | 0.18(0.06-0.57) | 0.29(0.09-0.89) | 0.70(0.24-2.08) | 1.02(0.20-5.12) | 0.10(0.04-0.26) | 0.90(0.49-1.68) | Tralokinumab 300 mg | . | . |
| 0.49(0.24-0.98) | 0.55(0.27-1.09) | 0.20(0.09-0.44) | 0.33(0.15-0.72) | 0.52(0.24-1.13) | 1.26(0.61-2.58) | 1.83(0.45-7.38) | 0.19(0.12-0.29) | 1.63(0.58-4.58) | 1.80(0.64-5.06) | Upadacitinib 15 mg | 0.63(0.41-0.97) |
| 0.31(0.16-0.63) | 0.35(0.17-0.70) | 0.13(0.06-0.28) | 0.21(0.10-0.46) | 0.34(0.16-0.72) | 0.81(0.39-1.66) | 1.18(0.29-4.74) | 0.12(0.08-0.19) | 1.04(0.37-2.94) | 1.16(0.41-3.25) | 0.64(0.44-0.94) | Upadacitinib 30 mg |

**Table S5. Network meta-analysis in comparing different interventions for EASI-90.**

| Abrocitinib 100 mg | 0.73(0.40-1.31) | . | . | . | . | . | 3.22(1.64-6.32) | . | . | . | . |
| --- | --- | --- | --- | --- | --- | --- | --- | --- | --- | --- | --- |
| 0.73(0.44-1.20) | Abrocitinib 200 mg | . | . | . | . | . | 4.43(2.28-8.61) | . | . | . | . |
| 3.45(1.64-7.26) | 4.75(2.27-9.96) | Baricitinib 1 mg | 0.47(0.23-0.96) | 0.31(0.15-0.60) | . | . | 0.93(0.43-2.03) | . | . | . | . |
| 1.63(0.79-3.36) | 2.25(1.09-4.62) | 0.47(0.29-0.77) | Baricitinib 2 mg | 0.65(0.36-1.16 | . | . | 1.97(0.99-3.95) | . | . | . | . |
| 1.05(0.51-2.16) | 1.45(0.71-2.96) | 0.31(0.19-0.49) | 0.65(0.41-1.01) | Baricitinib 4 mg | . | . | 3.06(1.57-5.95) | . | . | . | . |
| 0.35(0.15-0.83) | 0.48(0.20-1.14) | 0.10(0.04-0.24) | 0.21(0.09-0.49) | 0.33(0.14-0.76) | Dupilumab 300 mg | . | 9.23(4.66-18.30) | . | . | . | . |
| 3.30(0.70-15.54) | 4.54(0.97-21.35) | 0.96(0.20-4.47) | 2.02(0.44-9.35) | 3.13(0.68-14.45) | 9.46(1.90-47.14) | Nemolizumab 30 mg | 0.98(0.23-4.17) | . | . | . | . |
| 3.22(1.89-5.51) | 4.43(2.60-7.55) | 0.93(0.56-1.56) | 1.97(1.22-3.20) | 3.06(1.90-4.92) | 9.23(4.66-18.30) | 0.98(0.23-4.17) | Placebo | 0.18(0.06-0.57) | 0.21(0.07-0.65) | 0.14(0.07-0.27) | 0.05(0.03-0.10) |
| 0.60(0.22-1.64) | 0.82(0.30-2.25) | 0.17(0.06-0.47) | 0.36(0.14-0.98) | 0.56(0.21-1.51) | 1.71(0.57-5.12) | 0.18(0.03-0.98) | 0.18(0.08-0.44) | Tralokinumab 150 mg | 1.13(0.55-2.34) | . | . |
| 0.67(0.24-1.86) | 0.93(0.34-2.55) | 0.20(0.07-0.53) | 0.41(0.15-1.11) | 0.64(0.24-1.71) | 1.93(0.64-5.80) | 0.20(0.04-1.11) | 0.21(0.09-0.50) | 1.13(0.59-2.19) | Tralokinumab 300 mg | . | . |
| 0.42(0.20-0.88) | 0.58(0.28-1.21) | 0.12(0.06-0.25) | 0.26(0.13-0.52) | 0.40(0.20-0.80) | 1.20(0.51-2.82) | 0.13(0.03-0.59) | 0.13(0.08-0.22) | 0.70(0.26-1.91) | 0.62(0.23-1.69) | Upadacitinib 15 mg | 0.45(0.29-0.68) |
| 0.18(0.09-0.38) | 0.25(0.12-0.52) | 0.05(0.03-0.11) | 0.11(0.05-0.22) | 0.17(0.08-0.35) | 0.52(0.22-1.22) | 0.05(0.01-0.26) | 0.06(0.03-0.09) | 0.30(0.11-0.83) | 0.27(0.10-0.73) | 0.43(0.29-0.64) | Upadacitinib 30 mg |

**Table S6. Network meta-analysis in comparing different interventions for EASI-75.**

| Abrocitinib 100 mg | 0.85(0.45-1.60) | . | . | . | . | . | . | . | 3.07(1.67-5.64) | . | . | . | . |
| --- | --- | --- | --- | --- | --- | --- | --- | --- | --- | --- | --- | --- | --- |
| 0.85(0.51-1.41) | Abrocitinib 200 mg | . | . | . | . | . | . | . | 3.63(1.97-6.70) | . | . | . | . |
| 3.04(1.62-5.68) | 3.59(1.92-6.72) | Baricitinib 1 mg | 0.71(0.42-1.21) | 0.43(0.26-0.73) | . | . | . | . | 1.01(0.59-1.73) | . | . | . | . |
| 2.17(1.16-4.04) | 2.56(1.37-4.78) | 0.71(0.49-1.04) | Baricitinib 2 mg | 0.60(0.36-1.01) | . | . | . | . | 1.42(0.84-2.40) | . | . | . | . |
| 1.31(0.70-2.43) | 1.55(0.83-2.88) | 0.43(0.30-0.62) | 0.60(0.42-0.87) | Baricitinib 4 mg | . | . | . | . | 2.35(1.39-3.97) | . | . | . | . |
| 0.35(0.14-0.91) | 0.41(0.16-1.07) | 0.12(0.05-0.28) | 0.16(0.07-0.39) | 0.27(0.11-0.65) | Delgocitinib 0.25% | 0.62(0.24-1.62) | . | . | 9.58(3.80-24.19) | . | . | . | . |
| 0.24(0.08-0.70) | 0.28(0.10-0.83) | 0.08(0.03-0.22) | 0.11(0.04-0.31) | 0.18(0.07-0.51) | 0.68(0.30-1.56) | Delgocitinib 0.5% | . | . | 10.67(2.73-41.60) | . | . | . | . |
| 0.46(0.23-0.92) | 0.55(0.28-1.09) | 0.15(0.08-0.28) | 0.21(0.12-0.39) | 0.36(0.20-0.65) | 1.33(0.52-3.39) | 1.95(0.67-5.65) | Dupilumab 300 mg | . | 6.61(4.13-10.58) | . | . | . | . |
| 1.75(0.59-5.20) | 2.07(0.70-6.15) | 0.58(0.20-1.63) | 0.81(0.29-2.28) | 1.34(0.48-3.77) | 5.00(1.42-17.64) | 7.34(1.89-28.55) | 3.76(1.28-11.03) | Nemolizumab 30 mg | 1.76(0.67-4.62) | . | . | . | . |
| 3.07(1.86-5.07) | 3.63(2.20-6.00) | 1.01(0.69-1.47) | 1.42(0.98-2.06) | 2.35(1.62-3.41) | 8.78(3.91-19.73) | 12.89(4.97-33.47) | 6.61(4.13-10.58) | 1.76(0.67-4.62) | Placebo | 0.17(0.07-0.43) | 0.18(0.07-0.45) | 0.11(0.07-0.18) | 0.05(0.03-0.09) |
| 0.52(0.22-1.26) | 0.62(0.26-1.49) | 0.17(0.08-0.39) | 0.24(0.11-0.54) | 0.40(0.18-0.90) | 1.50(0.51-4.42) | 2.20(0.67-7.26) | 1.13(0.48-2.66) | 0.30(0.09-1.00) | 0.17(0.08-0.35) | Tralokinumab 150 mg | 1.04(0.56-1.94) | . | . |
| 0.54(0.23-1.31) | 0.64(0.27-1.55) | 0.18(0.08-0.40) | 0.25(0.11-0.56) | 0.42(0.18-0.94) | 1.55(0.53-4.59) | 2.28(0.69-7.53) | 1.17(0.49-2.76) | 0.31(0.09-1.04) | 0.18(0.09-0.36) | 1.04(0.59-1.82) | Tralokinumab 300 mg | . | . |
| 0.32(0.17-0.61) | 0.38(0.20-0.72) | 0.11(0.06-0.18) | 0.15(0.08-0.26) | 0.24(0.14-0.42) | 0.91(0.37-2.26) | 1.34(0.47-3.78) | 0.69(0.37-1.28) | 0.18(0.06-0.52) | 0.10(0.07-0.16) | 0.61(0.27-1.40) | 0.59(0.26-1.35) | Upadacitinib 15 mg | 0.59(0.37-0.95) |
| 0.18(0.09-0.34) | 0.21(0.11-0.41) | 0.06(0.03-0.10) | 0.08(0.05-0.14) | 0.14(0.08-0.24) | 0.51(0.20-1.27) | 0.75(0.26-2.12) | 0.38(0.20-0.72) | 0.10(0.04-0.29) | 0.06(0.04-0.09) | 0.34(0.15-0.79) | 0.33(0.14-0.76) | 0.56(0.37-0.83) | Upadacitinib 30 mg |

**Table S7. Network meta-analysis in comparing different interventions for EASI-50.**

| Abrocitinib 100 mg | 1.05(0.44-2.52) | . | . | . | . | 3.16(1.47-6.82) | . | . |
| --- | --- | --- | --- | --- | --- | --- | --- | --- |
| 1.05(0.53-2.07) | Abrocitinib 200 mg | . | . | . | . | 3.01(1.43-6.36) | . | . |
| 0.68(0.29-1.58) | 0.64(0.28-1.50) | Delgocitinib 0.25% | 1.46(0.54-3.94) | . | . | 4.71(2.54-8.75) | . | . |
| 1.00(0.37-2.69) | 0.95(0.36-2.55) | 1.48(0.70-3.16) | Delgocitinib 0.5% | . | . | 3.12(1.16-8.37) | . | . |
| 0.30(0.13-0.68) | 0.28(0.13-0.64) | 0.44(0.20-0.96) | 0.30(0.12-0.75) | Dupilumab 300 mg | . | 10.59(6.28-17.86) | . | . |
| 2.53(0.88-7.26) | 2.41(0.85-6.87) | 3.75(1.37-10.30) | 2.53(0.82-7.80) | 8.48(3.16-22.74) | Nemolizumab 30 mg | 1.25(0.54-2.88) | . | . |
| 3.16(1.67-5.99) | 3.01(1.61-5.65) | 4.69(2.66-8.26) | 3.16(1.49-6.72) | 10.59(6.28-17.86) | 1.25(0.54-2.88) | Placebo | 0.19(0.09-0.38) | 0.15(0.07-0.31) |
| 0.60(0.26-1.40) | 0.57(0.25-1.32) | 0.89(0.40-1.96) | 0.60(0.23-1.53) | 2.00(0.93-4.30) | 0.24(0.09-0.65) | 0.19(0.11-0.33) | Tralokinumab 150 mg | 0.80(0.45-1.40) |
| 0.48(0.20-1.11) | 0.45(0.20-1.05) | 0.71(0.32-1.57) | 0.48(0.19-1.22) | 1.60(0.74-3.43) | 0.19(0.07-0.52) | 0.15(0.09-0.26) | 0.80(0.49-1.30) | Tralokinumab 300 mg |

**Table S8. P-score rankings of IGA-0/1.**

| **Interventions** | **P-score** | **Rank** |
| --- | --- | --- |
| Upadacitinib 30 mg qd | 0.9414 | 1 |
| Delgocitinib 0.5% bid | 0.8827 | 2 |
| Upadacitinib 15 mg qd | 0.7890 | 3 |
| Delgocitinib 0.25% bid | 0.7380 | 4 |
| Tralokinumab 150 mg q2w | 0.6839 | 5 |
| Dupilumab 300 mg q4w | 0.5904 | 6 |
| Tralokinumab 300 mg q2w | 0.5825 | 7 |
| Baricitinib 4 mg qd | 0.4975 | 8 |
| Abrocitinib 200 mg qd | 0.3728 | 9 |
| Nemolizumab 30 mg q4w | 0.3066 | 10 |
| Abrocitinib 100 mg qd | 0.2871 | 11 |
| Baricitinib 2 mg qd | 0.2198 | 12 |
| Baricitinib 1 mg qd | 0.0747 | 13 |
| Placebo | 0.0337 | 14 |

**Table S9. P-score rankings of NRS-4.**

| **Interventions** | **P-score** | **Rank** |
| --- | --- | --- |
| Tralokinumab 300 mg q2w | 0.8447 | 1 |
| Upadacitinib 30 mg qd | 0.8157 | 2 |
| Nemolizumab 30 mg q4w | 0.8194 | 3 |
| Tralokinumab 150 mg q2w | 0.7988 | 4 |
| Dupilumab 300 mg q4w | 0.7137 | 5 |
| Upadacitinib 15 mg qd | 0.6065 | 6 |
| Abrocitinib 200 mg qd | 0.3839 | 7 |
| Baricitinib 4 mg qd | 0.3746 | 8 |
| Abrocitinib 100 mg qd | 0.3330 | 9 |
| Baricitinib 2 mg qd | 0.2074 | 10 |
| Baricitinib 1 mg qd | 0.0618 | 11 |
| Placebo | 0.0410 | 12 |

**Table S10. P-score rankings of EASI-90.**

| **Interventions** | **P-score** | **Rank** |
| --- | --- | --- |
| Upadacitinib 30 mg qd | 0.9926 | 1 |
| Dupilumab 300 mg q4w | 0.8520 | 2 |
| Upadacitinib 15 mg qd | 0.8022 | 3 |
| Tralokinumab 150 mg q2w | 0.6716 | 4 |
| Tralokinumab 300 mg q2w | 0.6121 | 5 |
| Abrocitinib 200 mg qd | 0.6011 | 6 |
| Abrocitinib 100 mg qd | 0.4460 | 7 |
| Baricitinib 4 mg qd | 0.4384 | 8 |
| Baricitinib 2 mg qd | 0.2733 | 9 |
| Nemolizumab 30 mg q4w | 0.1291 | 10 |
| Placebo | 0.1018 | 11 |
| Baricitinib 1 mg qd | 0.0797 | 12 |

**Table S11. P-score rankings of EASI-75.**

| **Interventions** | **P-score** | **Rank** |
| --- | --- | --- |
| Upadacitinib 30 mg qd | 0.9707 | 1 |
| Delgocitinib 0.5% bid | 0.8850 | 2 |
| Upadacitinib 15 mg qd | 0.8098 | 3 |
| Delgocitinib 0.25% bid | 0.7613 | 4 |
| Dupilumab 300 mg q4w | 0.6680 | 5 |
| Tralokinumab 150 mg q2w | 0.6266 | 6 |
| Tralokinumab 300 mg q2w | 0.6098 | 7 |
| Abrocitinib 200 mg qd | 0.4579 | 8 |
| Abrocitinib 100 mg qd | 0.3915 | 9 |
| Baricitinib 4 mg qd | 0.3097 | 10 |
| Nemolizumab 30 mg q4w | 0.2303 | 11 |
| Baricitinib 2 mg qd | 0.1759 | 13 |
| Baricitinib 1 mg qd | 0.0548 | 14 |
| Placebo | 0.0488 | 15 |

**Table S12. P-score rankings of EASI-50.**

| **Interventions** | **P-score** | **Rank** |
| --- | --- | --- |
| Dupilumab 300 mg q4w | 0.9776 | 1 |
| Tralokinumab 300 mg q2w | 0.8246 | 2 |
| Tralokinumab 150 mg q2w | 0.6855 | 3 |
| Delgocitinib 0.25% bid | 0.6380 | 4 |
| Abrocitinib 100 mg qd | 0.4203 | 5 |
| Delgocitinib 0.5% bid | 0.4177 | 6 |
| Abrocitinib 200 mg qd | 0.3921 | 7 |
| Nemolizumab 30 mg q4w | 0.1065 | 8 |
| Placebo | 0.0379 | 9 |

**Fig S1. Forest plots of efficacy outcomes (comparison with placebo).**

**
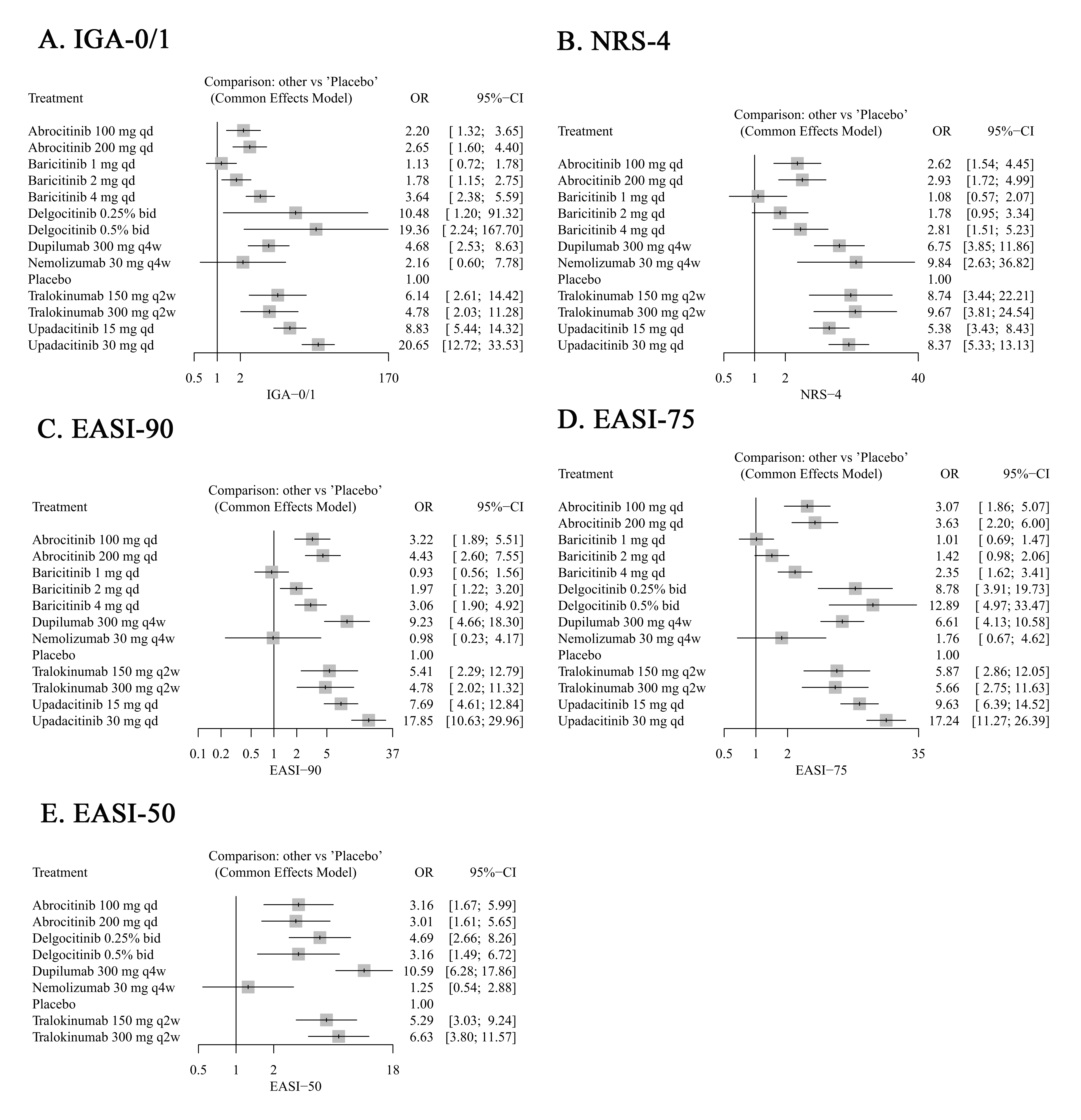
**

**Fig S2. Forest plots of safety outcomes (comparison with dupilumab 300 mg q4w).**

**
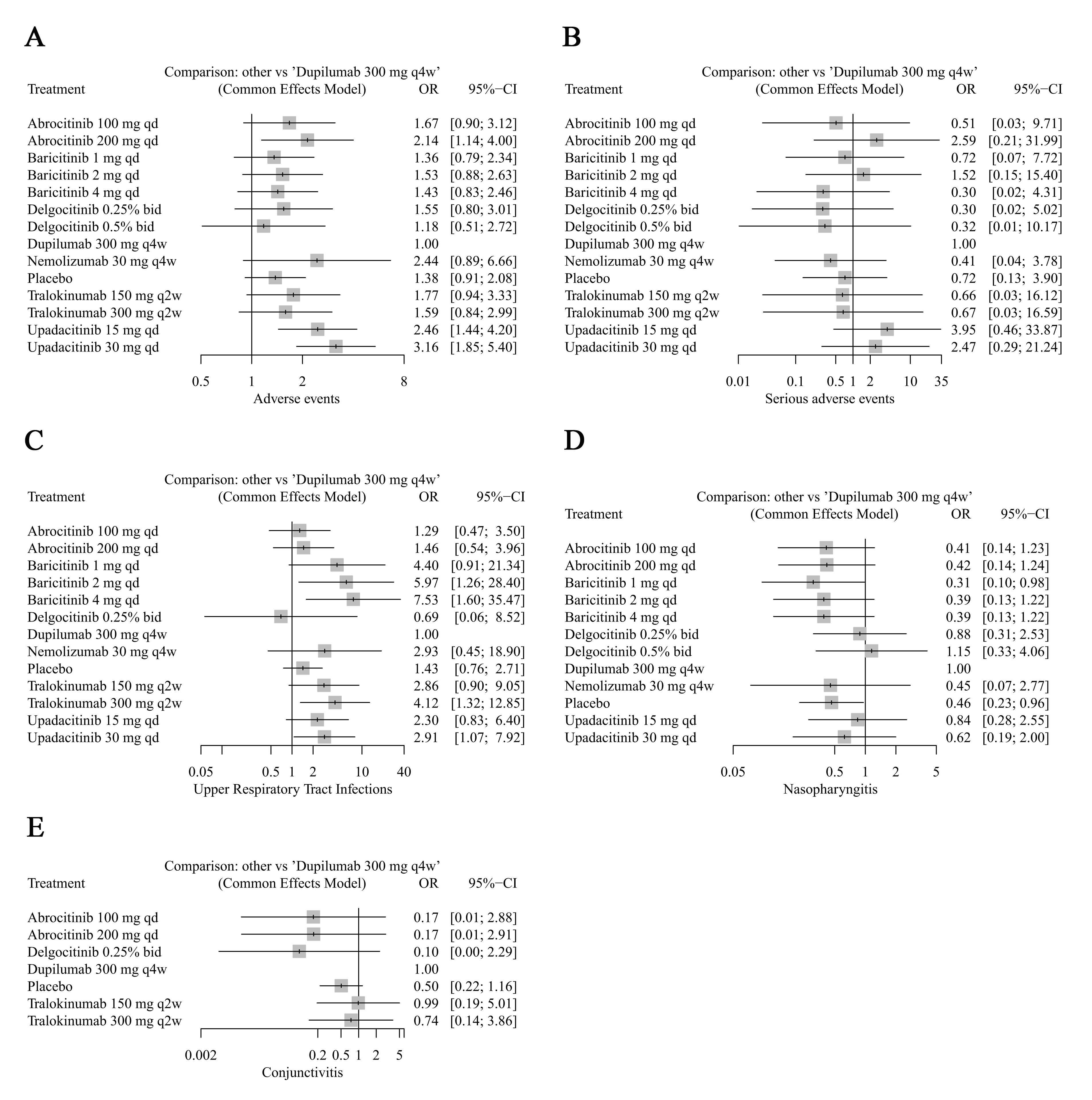
**

**Fig S3. Sensitivity analysis - Forest plots of combined therapy for efficacy outcomes (comparison with dupilumab 300 mg q4w).**


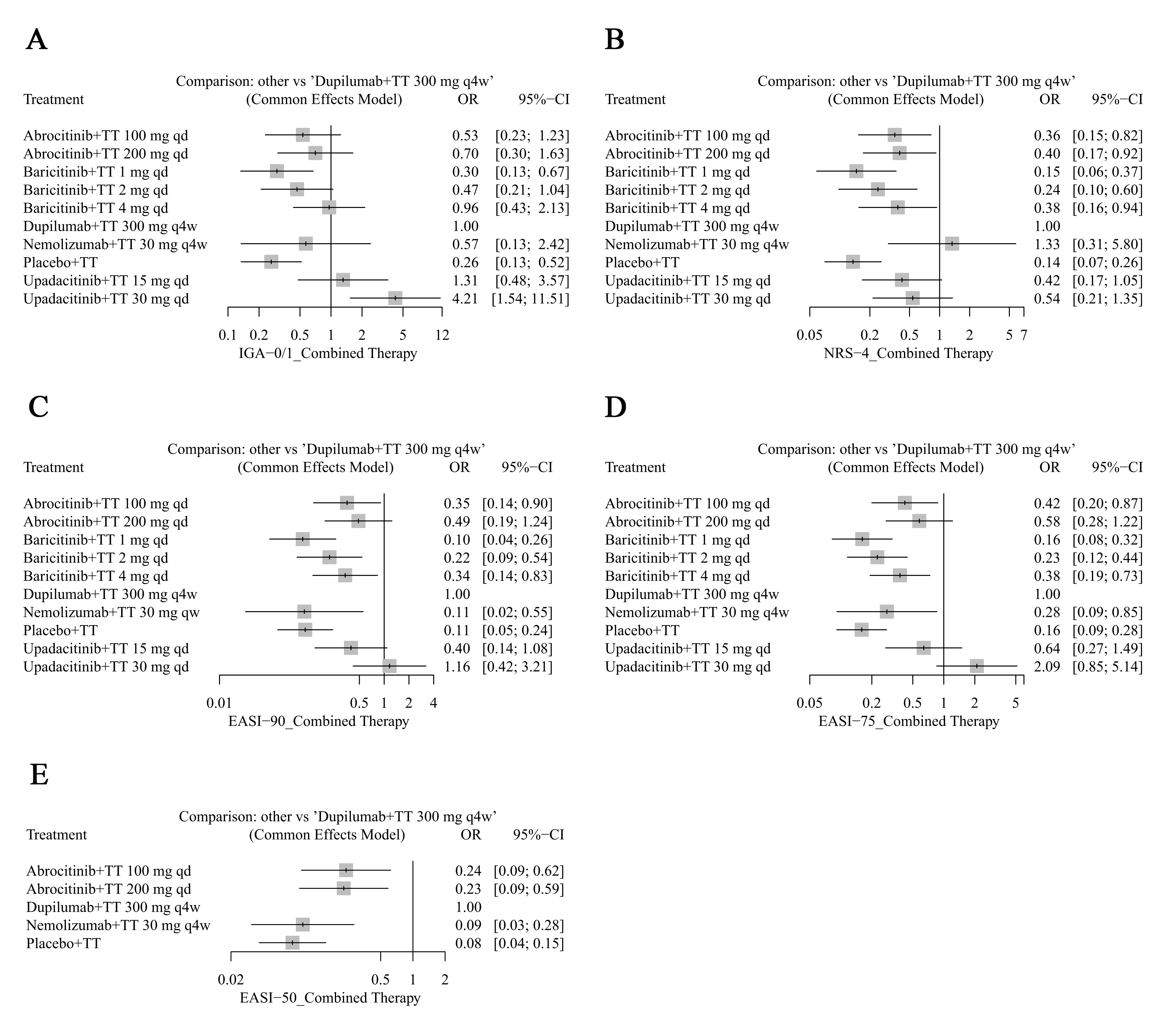


*TT=Topical therapies

**Fig S4. Sensitivity analysis - Forest plots of monotherapy for efficacy outcomes (comparison with dupilumab 300 mg q4w).**


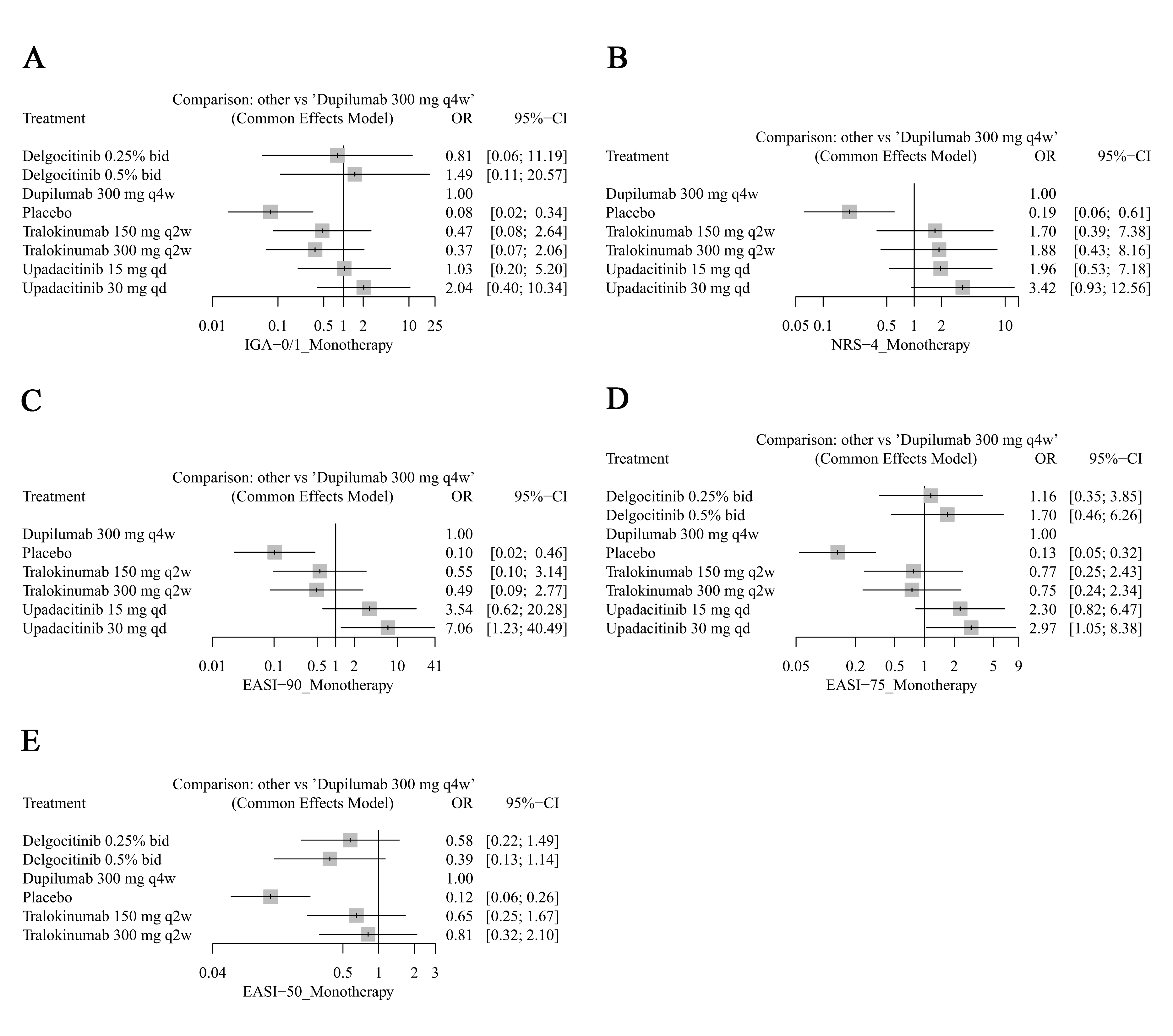


**Fig S5. Sensitivity analysis - Forest plots of combined therapy for safety outcomes (comparison with dupilumab 300 mg q4w).**


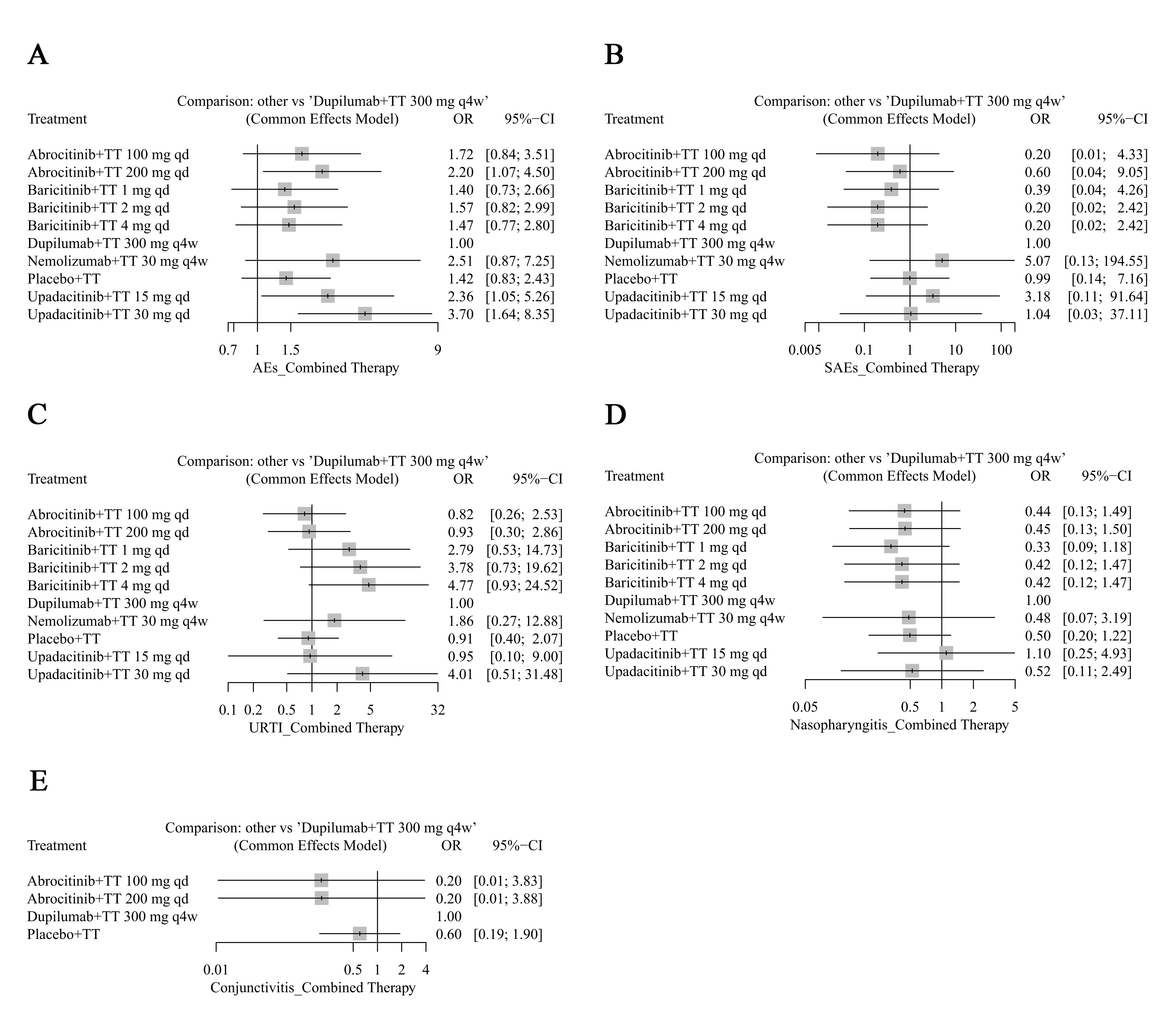


*TT=Topical therapies

**Fig S6. Sensitivity analysis - Forest plots of monotherapy for safety outcomes (comparison with dupilumab 300 mg q4w).**


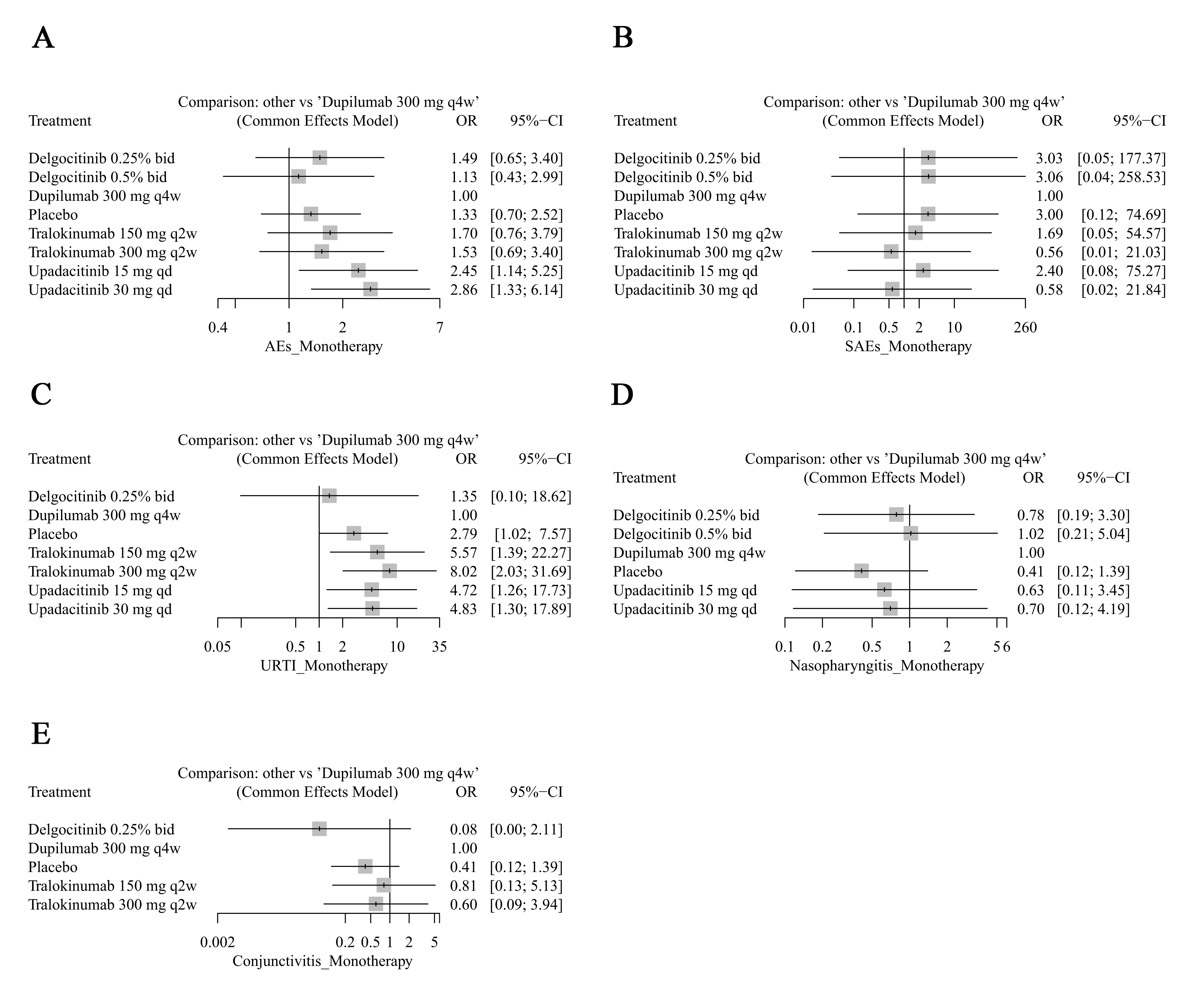

Supplement: S1 File — Table S1. Search Strategy. Table S2. Heterogeneity assessment. Table S3–S7. Network meta-analysis in comparing different interventions. Table S8–S12. P-score rankings results. Fig S1. Forest plots of efficacy outcomes (comparison with placebo). Fig S2. Forest plots of safety outcomes (comparison with dupilumab 300 mg q4w). Fig S3–S6 Forest plots of sensitivity analysis. (DOCX) [file pone.0319400.s001.docx]
